# RIGOR Guidelines: Escalating STAIR and STEPS for Effective Translational Research

**DOI:** 10.1007/s12975-012-0209-2

**Published:** 2012-09-05

**Authors:** Paul A. Lapchak, John H. Zhang, Linda J. Noble-Haeusslein

**Affiliations:** 1 Department of Neurology, Cedars-Sinai Medical Center, Davis Research Building, D-2091, 110 N. George Burns Road, Los Angeles, CA 90048 USA; 2 Department of Neurosurgery, Anesthesiology, Neurology, and Physiology and Pharmacology, Loma Linda University School of Medicine, Loma Linda, CA 92350 USA; 3 Department of Neurological Surgery, Physical Therapy and Rehabilitation Science, and Brain and Spinal Injury Center (BASIC), University of California, San Francisco, Box 0112, 513 Parnassus Avenue, HSE-722, San Francisco, CA 94143-0112 USA

**Keywords:** Translational, Brain, Stroke, Hemorrhage, SAH, ICH, Clinical Trial, NIHSS, STAIR, STEPS, RIGOR

## Abstract

Stroke continues to be a serious and significant health problem in the USA and worldwide. This article will emphasize the need for good laboratory practices, transparent scientific reporting, and the use of translational research models representative of the disease state to develop effective treatments. This will allow for the testing and development of new innovative strategies so that efficacious therapies can be developed to treat ischemic and hemorrhagic stroke. This article recommends guidelines for effective translational research, most importantly, the need for study blinding, study group randomization, power analysis, accurate statistical analysis, and a conflict of interest statement. Additional guidelines to ensure reproducibility of results and confirmation of efficacy in multiple species are discussed.

“We can’t solve problems by using the same kind of thinking we used when we created them.”

Albert Einstein (German born American Physicist. 1879–1955)

## Translational Research is Key to Drug Development Success

There remains a critical medical need for new therapeutic strategies to treat acute ischemic stroke (AIS) and hemorrhage stroke, including subarachnoid hemorrhage (SAH) and intraventricular hemorrhage (IVH), to improve the quality of life for stroke victims. This article will emphasize the need for good laboratory practices, transparent scientific reporting, and the use of translational research models representative of the disease state, with bonafide clinically relevant endpoints to develop and test new innovative strategies so that efficacious therapies can be developed to treat stroke, which remains a serious and significant health problem. According to the US stroke statistics, each year, approximately 795,000 victims suffer a new or recurrent stroke, 75 % of which are first strokes. Eighteen percent of stroke victims (143,100) die from the brain attack. AIS is now the fourth leading cause of death and the leading cause of adult disability in the USA with an estimated cost range of $69–74 billion annually [1–3], a cost that continues to increase due to the high cost of medical insurance plans and care in the USA. Currently, the only Food and Drug Administration (FDA)-approved treatment for stroke is the thrombolytic tissue plasminogen activator (tPA) if administered within 3 h of a stroke. However, the clot-busting drug therapeutic window has been expanded and has been shown to be beneficial if given 3–6 h after a stroke [4–6]. Although thrombolysis is now widely accepted as a standard of care for AIS, only a minority (5 %) of AIS patients are treated with tPA in the USA [7].

Importantly, a recent cost-effectiveness survey based upon the utilization of tPA, the only FDA-approved treatment for AIS [3] within 3–4.5 h after stroke onset, clearly showed that in AIS patients with National Institutes of Health Stroke Scale (NIHSS) scores of 0–19, there was an incremental benefit to the patient administered with tPA compared to patients with no treatment. Thus, there was benefit in terms of quality-adjusted life-years. However, interestingly, the analysis showed reduced benefit in patients with an NIHSS score >19, and there was no benefit in diabetic patients or patients with atrial fibrillation. Information gained from the repeated testing of tPA [4, 8], and subsequent population analyses should be incorporated into current and future translational development programs.

“The true sign of intelligence is not knowledge but imagination.”

Albert Einstein (German born American Physicist. 1879–1955)

It is clear that without rigorous translational research, which is based upon incremental prior knowledge, progress cannot be made. The basis for future translational research should incorporate novel ideas and a series of specified guidelines to increase the possibility of success in clinical trials and in the open heterogeneous patient population after the FDA approval process.

## A Brief Chronological History of Stroke

Historically, stroke, or a condition referred to as apoplexy, or the sudden onset of paralysis was first “reported” by Hippocrates over 2,400 years ago between 460 and 370 BC and can be found in Hippocratic transcripts [9]. In the mid to late 1500 s through the early 1600 s, there were many descriptions of apoplexy (apoplectic seizure) by Wepfer who correctly presented a realistic classification of apoplexy into hemorrhagic stroke and cerebral infarction in 1658 [10]. The fascinating account by Wepfer, discussed in detail by Gurdjian and Gurdjian [11], recounts the idea that body-derived “natural spirits” which become “vital spirits” are transported into the brain via the carotid and verterbal arteries and a network of arteries at the base of the brain (i.e., circle of Willis), which was quite elegantly described by Thomas Willis in the mid 1600 s [12]. So that the concept is not lost in translation, Wepfer’s suggestion implied that oxygenated blood was transported into the brain as a vital factor, as a source of nutrition, and Willis suggested that our current concept of the “cerebrovascular system” included a network of arteries, including the “circle of Willis”.

Autopsy records from the 1700 s confirmed two types of apoplexy, suggested to be serous apoplexy (apoplexia serosa) and sanguineous apoplexy (apoplexia sanguinea). Between the early 1600 s and the mid 1800 s, the 1866 writings of Dechambre summarized over 150 apoplexy references [13]. Subsequently, in 1856, Virchow [14] was the first physician to recognize that an embolus could result in a thromboembolism, and coined the terms related to the pathogenesis of ischemic stroke. In the more recent literature, Bramwell and Symonds published articles in 1886 [15] and 1924 [16], respectively, describing the “spontaneous” meningeal and SAH. It is now known that brain hemorrhage, SAH, ICH, and IVH occur in approximately 17–20 % of all patients with a stroke [1, 2, 17]. Hemorrhage is associated with a rapid decline and higher mortality rate than ischemic stroke. The 30-day mortality rate for ischemic stroke is estimated to be 8–12 %, whereas hemorrhagic stroke is estimated to be 5 0 % [18–21]. Both conditions are in dire need of treatment.

“If at first, the idea is not absurd, then there is no hope for it”

Albert Einstein (German born American Physicist. 1879–1955)

## Translational Stroke Research

The promise that translational studies will offer potentially significant treatment of AIS has still not been realized due to the lack of efficacy of neuroprotectants in randomized clinical trials, thus, the FDA has not approved any neuroprotectant strategy to treat stroke [22–25]. Moreover, neurorestorative molecules and stem cells that may promote recovery of function have not been developed to a point where they have been adequately tested in clinical trials, although this is a strategy where attention is justified [23, 25–27].

## The Status of STAIR

In 1999, the original set of stroke therapy academic industry roundtable (STAIR) recommendations, which resulted from a collaborative effort between academics and industry [28], documented a series of basic criteria or primary recommendations that should be followed to advance the field of stroke research. The STAIR report only addressed rodent and primate studies and their utility to measure at least two outcomes, functional response and infarct volume in the acute stroke phase (1–3 days) and thereafter (7–30 days). In contrast to STAIR, the report by Sharp and colleagues [29] emphasized the utility of the non-rodent species, *Oryctolagus cuniculus* (rabbit), to develop stroke therapies because of the track record and historical significance of the model in the preclinical development of tPA (see [22, 29] for references and discussion on this topic). The reader is also referred to a recent article by Cook and Tymianski that attempts to provide additional justification for the use of nonhuman primates to bridge the translational gap between animals and humans [30].

STAIR 1 also recommended the following:

1. Randomized and blinded studies
2. Efficacy in two or more laboratories
3. Replication in a second species
4. Consideration of sex difference
5. Consideration of route of administration (to administer intravenous or not)
6. Consideration of a clinically useful therapeutic window and dose response

From 2004 to 2011, STAIR continued to report on recommendations for developing neuroprotective therapies, expanding treatment options, utilizing combination therapies and designing clinical trials [31–34]. The recommendations attempted to address the problems resulting from the devastating failure of NXY-059 [35]. While NXY-059 was deemed an acceptable but not optimal development plan [36–40], by original STAIR criteria, retrospective analysis showed that its beneficial effects were over estimated in preclinical studies [36]. Thus, even though these new criteria were put into place, subsequent clinical trials, relying on this more stringent development criteria, failed to produce any benefit.

The 2009 STAIR report [33] included updated recommendations related to the conduct of good science or good laboratory practice. The most important aspects are the following:

1. Eliminating randomization and assessment bias
2. Defining inclusion/exclusion criteria
3. Conducting full power analysis and sample size calculations
4. Disclosing potential conflicts of interest

The recommendations also attempted to address comorbidities that are commonly recognized in stroke patients and the need for inclusion of those comorbidities in preclinical investigations. Thus, the following STAIR suggestion was made: “*after initial evaluations in young, healthy male animals, further studies should be performed in females, aged animals, and animals with co-morbid conditions such as hypertension, diabetes, and hypercholesterolemia*”. This is a recommendation for the use of an extensive number of animal models for future translational studies, in an attempt to effectively translate preclinical studies into the clinic, even though the development process for a neuroprotective has still not been validated by a “positive” clinical trial.

Nevertheless, understandably, the recommendation was centered around the idea that studies in animal models with comorbidities would better reproduce the “pathophysiological” state of patients presenting with strokes, but, clearly, this may not be the case due to the observations made below.

Using the National Institure of Neurological Disorders and Stroke (NINDS) rt-PA and ECASSIII trials as examples, the stroke patient population had the characteristics described in Table 1. The most pertinent aspects are that strokes occur in a mixed gender aged population, particularly those with a history of hypertension or diabetes, which is usually controlled by one or more pharmaceuticals. Moreover, the majority of stroke patients are not antihypertensive-naive [41, 42], and may receive anticoagulants and statins [41]. These points are important when considerations are made regarding the use of a “correct” animal model for drug development.

**Table 1:** Stroke patient characteristics

Characteristic	NINDS rt-PA(8)	ECASS III(4)
Age (years)	>66	>64.9
Gender (% male)	57–60	57.3–63.2
Mean NIHSS		10.7–11.6
Hypertension (%)	64–66	62.4–62.8
Diabetes (%)	20–24	14.8–16.6
Prior use of drugs (%)		
Aspirin/antiplatelet	26–40	31.1–32.5
Tobacco (current)	–	28.8–30.6
Tobacco (Ex-smoker)	–	20.6–24.6
Tobacco (current or Ex-smoker)	27–43	–

There is also significant information available in the literature regarding the group of refractory diabetic patients enrolled in the NINDS rt-PA trial [8] and the ECASS III trial [4], which includes a small percentage of patients with diabetes (Table 1). It is extremely difficult to treat diabetic stroke patients since they do not respond [3] or have an attenuated response to standard dose thrombolytic therapy [43–45]. Patients with diabetes have been shown to be independently associated with poor neurological outcome and higher mortality in the absence of thrombolytic treatment [43–45], and among patients treated with intravenous tPA [8], the presence of diabetes significantly reduces the odds of favorable outcome at 3 months [17].

Considering the information provided above and the refractory phenomenon in diabetic patients, will using a standard naive-hypertensive rodent be sufficient to predict drug efficacy in a heterogeneous population of stroke patients? Should translational studies attempt to address the diabetic population presenting with a stroke, or should “proof of concept” efficacy first be obtained for the larger mixed gender aged population, leaving clinical study in diabetics for post FDA drug approval?

## STEPS Toward a Stroke Therapy

The stem cell therapies as an emerging paradigm in stroke (STEPS) investigative team has paralleled the STAIR committee by documenting recommendation for developing stem cell therapy for stroke [46, 47]. STEPS is focused on a cell-based restorative therapy, whether the mechanism of action be central or peripheral, that may be independent of cell access to the ischemic penumbra or core. Many of the recommendation of STEPS are similar to STAIR recommendations including:

1. Test therapy in multiple strains
2. Replication in a second species
3. Consideration of age and gender
4. Functional outcome (minimum of 1 month)
5. Consideration of a clinically useful therapeutic window and dose response
6. Consideration of route of administration (intracerebral or systemic)
7. Study ischemic and hemorrhagic stroke subgroups

The second STEPS report [47] added little to the first report recommendations with respect to procedural issues, with the following exceptions:

1. Reproducibility (confirmation) in multiple laboratories and in two species
2. The possible addition of comorbidities (hypertensive, diabetic)
3. “Positive, neutral, and negative” study outcomes should be reported

## The RIGORs of Stroke Drug Development

The NINDS sponsored workshop on Improving the Quality of NINDS-Supported Preclinical and Clinical Research through Rigorous Study Design and Transparent Reporting was held in June, 2012 in Washington, DC [48]. The objective of the workshop was to improve the rigorous of preclinical research even though application of fundamental principles of experimental design and transparency in reporting are currently the standard of practice by the clinical research community.

Dr. Malcolm Macleod (Centre for Clinical Brain Sciences, University of Edinburgh) presented a PowerPoint session that is directly pertinent to translational stroke research (Please see original articles for additional information [49, 50]). Citing the Collaborative Approach to Meta-Analysis and Review of Animal Data from Experimental Studies projects, Macleod showed data to support the following conclusions:

1. Measured effect (improvement) was larger in studies done by investigators when there was no randomization.
2. Measured effect (improvement) was larger in studies done by investigators when there was no blinded assessment.
3. In stroke research, only 36 % of published studies reported randomization.
4. In stroke research, only 29 % of published studies were blinded.
5. Power analysis was only documented by 3 % of published studies.
6. There was no significant association (*r* ^2^ = 0.06) between the quality of science and the impact factor of the journal.
7. There was no significant association (*r* ^2^ = 0.004) between the quality of science and the number of citations of a particular study.
8. A significant amount of unpublished negative or neutral data cause an overestimation of efficacy because of published positive data.

Based upon the Macleod presentation and others based upon the following criteria listed below, there were few recurring themes for advancement of translational research, which should be incorporated into translational grant applications and publications reporting translational research, independent of the source of funding. This will ensure reproducible, valid research on an international level.

1. Experimental design
  - Rationale for the selected models and endpoints (animal and/or cellular)
  - Adequacy of the controls
  - Route and timing of intervention delivery/dosing
  - Justification of sample size, including power calculation
  - Statistical methods used in analysis and interpretation of results
  For grant applications, the investigator must present a solid and justified Experimental design including full power analysis for the sample size to be included in each experimental group. If there are multiple endpoints to be measured, the investigator should indicate how the study was powered and for what specific endpoint. A recommendation is made to present this information in manuscript submissions for stroke-related journals including Translational Stroke Research.
2. Minimizing bias
  - Methods of blinding (allocation concealment and blinded assessment of outcome)
  - Strategies for randomization and/or stratification
  - Reporting of data missing due to attrition or exclusion
  - Reporting of all results (negative and positive)
  Macleod showed correlative data for greater efficacy results when the investigator(s) were neither blinded or study randomized, thus reinforcing the importance of adequate randomization and blinding to minimize bias. This will require at least one member of the experimental team to remain naive to the study design, and the investigator charged with drug administration cannot be the person to randomize and blind the study. Moreover, to maintain blinding, the investigator responsible for endpoint determination (i.e., behavioral analysis) should be naive to the experimental groups and order/randomization of drug administration. A recommendation is made to use websites such as http://randomizer.org/ to produce randomization tables for all groups to be included in the study once power analysis for the number of animals per experiment (i.e., *n* or *N*) is established. For all studies, the investigator is encouraged to report to NIH in progress reports and in primary scientific publications all data including missing data due to attrition or exclusion, including technical complications.
3. Results
  - Independent validation/replication, if available
  - Robustness and reproducibility of the observed results
  - Dose–response and therapeutic window results
  In translational grant applications with the specific aim of filing an IND application to initiate a clinical trial, the investigator should incorporate studies to replicate findings in a second external laboratory and if possible, in a second species. This strategy will ensure robust reproducible results. All external studies should also be conducted in a blinded and randomized manner. Standard good laboratory practice should be followed for all pharmacological studies using standard dose–response analysis and therapeutic window analysis appropriate for the condition to be treated.
4. Interpretation of results
  - Alternative interpretations of the experimental data
  - Discussion of effect size in relation to potential clinical impact
  - Potential conflicts of interest

While most pharmacological studies aimed at developing a therapy are easily interpreted with a simplistic endpoint that is being measured such as behavioral improvement or infarct reduction, there are some instances where the investigator should be prepared to offer alternative explanations of their results. For example, should reduced infarct volume in a rodent model of embolic stroke, in the absence of behavioral improvement significant, be used to establish an IND? Alternatively, if there is significant behavioral improvement without infarct volume reduction, can this observation lead to a stroke clinical trial where NIHSS and modified Rankin Scale are used as primary endpoints [4, 8, 35, 51–53]? The investigator should also incorporate a discussion of their findings in the context of the bigger picture of the disease being studied, and whether their justified endpoint is clinically relevant.

It’s not that I’m so smart, it’s just that I stay with problems longer.

Albert Einstein (German born American Physicist. 1879–1955)

## Conclusion

In conclusion, the following recommendations are made to investigators interested in conducting translational research using good laboratory practices. The RIGOR guidelines discussed above, most importantly, method of blinding, study group randomization, complete power analysis and statistical analysis, should be incorporated into translational grant applications and are recommended for all manuscripts submitted to Translational Stroke Research. A conflict of interest statement is required for all investigators on the study to include all funding for the study, collaborations with the pharmaceutical or biotechnology industry, scientific or clinical advisory boards, and financial interest in the industry broadly related to current work. It is recommended that authors submitting articles to Translational Stroke Research include a checklist (see Table 2) indicating that the study was conducted using current guidelines.

**Table 2:** Author RIGOR Criteria Adherence

Guideline criteria	Yes	No
Randomized		
Blinded		
Power Analysis		
Statistical Analysis		
Justification of Model		
All data are being reported		
Conflict of Interest Statement		
